# Exercise Mimetics in Aging: Suggestions from a Systematic Review

**DOI:** 10.3390/nu17060969

**Published:** 2025-03-10

**Authors:** Emiliana Giacomello, Claudio Nicoletti, Marta Canato, Luana Toniolo

**Affiliations:** 1 Department of Medicine, Surgery and Health Sciences, University of Trieste, 34149 Trieste, Italy; 2 Department of Molecular and Developmental Medicine, University of Siena, 53100 Siena, Italy; claudio.nicoletti@unisi.it; 3 Laboratory of Muscle Biophysics, Department of Biomedical Sciences, University of Padova, 35131 Padova, Italy; marta.canato@unipd.it

**Keywords:** exercise, exercise mimetics, metabolism, nutrient sensing pathway, myokines, aging, lifespan, health span, natural compounds

## Abstract

**Background/Objectives:** Growth in the aging world population is accompanied by an increase in comorbidities, profoundly impacting the quality of life of older people. This development has motivated a large effort to investigate the mechanisms underlying aging and the search for countermeasures. The most investigated strategies envisage the control of diet and physical exercise, which exploit both common and distinct mechanisms to promote health. Since the application of nutritional and exercise protocols to aged persons introduces several issues due to their disabled state, some strategies have been developed. The nutritional approach exploits a wide range of compounds, including calorie restriction mimetics, supplements, antioxidants, and others. In the context of exercise, in recent years, molecules able to provide similar effects to exercise, the so-called exercise mimetics, have been developed. **Methods:** To have a better perspective on exercise mimetics and their connection with nutrition, we performed a systematic search of the PubMed and Scopus databases using the term “exercise mimetics”. **Results:** In total, 97 research articles were selected and discussed. The present review provides evidence of the presence of multiple exercise-mimetic compounds and physical strategies that can target metabolic pathways, oxidative stress defense mechanisms, or myokine modulation. **Conclusions:** Interestingly, this review highlights that an important number of exercise mimetics are represented by products of natural origin and supplements assimilable with diet. This evidence provides a further link between exercise and nutrition and confers a central role on nutrition in the context of exercise mimetics.

## 1. Introduction

Life expectancy has increased considerably over the last decades. However, the increase in lifespan is not always paralleled by an improved health span. Evidence for this is a source of great concern for the health system, which is facing an increase in a weaker population [1].

Aging is associated with important changes in body composition, such as a reduction in lean body mass and an increase in body fat, alterations in systemic metabolism [2,3], an imbalance in inflammatory response, and the establishment of a complex condition characterized by several deficits that can result in frailty [4,5]. Actually, the rise in life expectancy carries an intrinsic disadvantage, which consists of an increase in chronic diseases and complex situations and includes sarcopenia, bone weakness, cardiovascular dysfunction, diabetes, cancer, depression, and degenerative disorders [6]. In the light of a growing interest not only to increase the lifespan but also to improve the health span, the most investigated strategies envisage the control of diet and the application of exercise protocols, presenting both common and distinct mechanisms responsible for inducing a healthy status [7,8].

It has been largely demonstrated that diet has a relevant impact on healthy aging, which can be promoted by dietary diversity, the use of functional foods or supplements, and the application of calorie restriction regimens [7]. Analogously, physical activity is an essential factor in the primary and secondary prevention of premature death from any cause, such as cardiovascular disease, diabetes, some cancers, and osteoporosis [9], and can also have an impact on mood [10].

But how do diet and exercise have such an influence on so many and such diverse targets? The fact that skeletal muscle is the organ that uses most of the energy intake in performing physical activity places this tissue in a central position in the development of strategies to combat or slow the aging process. Concerning physical activity, there is a body of evidence showing that skeletal muscle strongly participates in the body response to exercise, providing a pivotal role for this tissue in the combat against aging and related pathologies [11]. Actually, in skeletal muscle, exercise induces a remodeling of the contractile apparatus, modification of the neuromuscular junction, metabolic adaptation of myofibers, proliferation of satellite cells, modulation of the capillary bed, and the production of myokines, which are released into the blood stream and are then able to interact with other organs [11,12]. Either via autocrine, paracrine, or endocrine signaling, myokines establish multi-organ crosstalk. In this way, exercise induces multiple modifications that involve molecular, biochemical, and physiological mechanisms of communication. As a consequence, exercise positively impacts the form and function of bone, the brain, the liver, the gastrointestinal tract, the immune system, and other areas [11,13]. Although myokines are key actors in keeping all organs in communication during exercise (the so-called body adaptation to exercise), most probably, exercise can also directly influence some organs and their functions independently of skeletal muscle mediation.

Considering that aging entails dysfunctions at multi-organ level, and exercise has a multi-organ positive impact, exercise represents a preferential route to combat and/or slow the aging process and to prevent and/or decrease the consequences of comorbidities. However, although it is possible for young and middle-aged people to follow exercise protocols, aged persons cannot always engage in exercise interventions due to their disabled state. This induces the exploration of new strategies able to provide similar effects to exercise. We define exercise mimetics as those agents that simulate or improve the effects of exercise [14].

In the present manuscript, we report the results of a systematic review of the literature on the effects of exercise mimetics on skeletal muscle and other organs. Exercise mimetics have been shown to interact with one or more pathways, improving various aspects of different organs’ physiology. Therapeutic agents have been grouped into three categories—those that regulate metabolic pathways, those that participate in myokine pathways, and physical approaches—and discuss them according to the data in the literature. Interestingly, this systematic review highlights that an important part of exercise mimetics is represented by products of natural origin and supplements assimilable with diet, providing a further link between exercise and nutrition and conferring a central role on nutrition in the context of exercise mimetics.

## 2. Materials and Methods

A systematic search of the PubMed and Scopus databases was performed up to November 2024 using the term “exercise mimetics”, following the Preferred Reporting Items for Systematic Reviews and Meta-Analyses (PRISMA) checklist guidelines [15].

**Figure 1:**
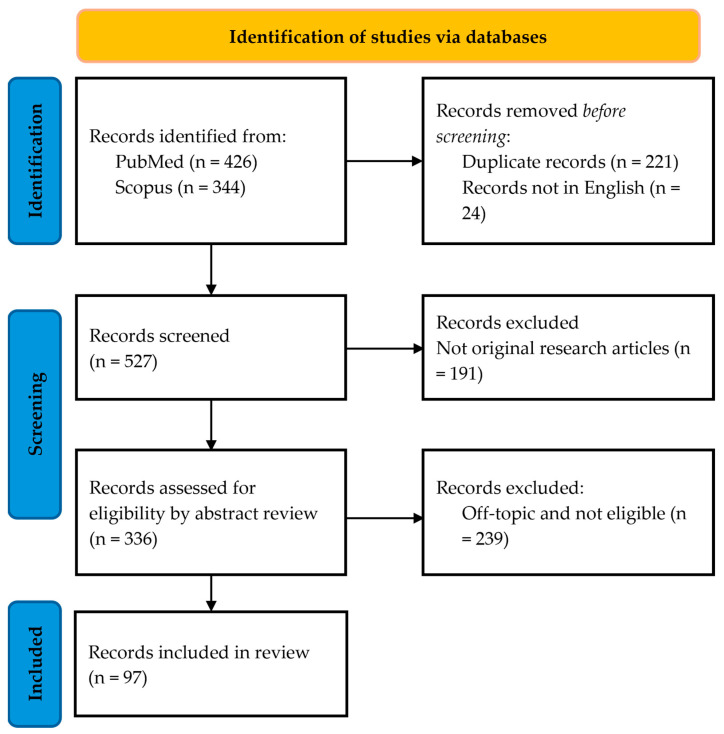
PRISMA flow diagram of the systematic search (https://creativecommons.org/licenses/by/4.0/ accessed on 9 March 2025).

As reported in Figure 1, the search of the PubMed and Scopus databases produced 426 and 326 records, respectively. Duplicated records, reviews, editorials, and opinion articles were removed. Moreover, records not in English were excluded. Afterward, an analysis of the abstracts and texts led to the exclusion of 239 articles because they were not relevant to the topic of the literature search or did not satisfy the eligibility criteria. The eligibility criteria were determined with a PICOS (participants, interventions, comparison, outcomes, study design) approach with the following parameters:

Participants: humans, animal models, in vitro cell systems;
Interventions: application of exercise mimetics (both active compounds and physical methods);
Comparison: the study’s outcome parameters must have been measured pre-treatment and post-treatment or with and without treatment;
Outcomes: analysis of physical exercise capacity, systemic parameters, metabolic pathways, and molecular mechanisms;
Study design: randomized and non-randomized clinical trials, animal and in vitro studies, longitudinal and cross-sectional protocols.

Finally, 97 research articles were included in the writing of the current review (Table S1).

## 3. Results and Discussion

With the awareness that the effects of physical activity could involve multiple pathways inducing broad-ranging consequences in different tissues and organs [16], we report here the potential of exercise mimetics by including them in three major groups: those that regulate metabolic pathways (glucose metabolism, oxidative stress, and mitochondria), those that participate in myokine pathways, and physical/mechanical approaches. We report and discuss below the benefits observed in skeletal muscle and other organs and tissues. Table S1 reports the selected records, the therapeutic agents, the experimental models used to test them, the effects exerted, and their targets.

### 3.1. Metabolic Pathways

Metabolic deterioration is a common factor in aging. It accompanies several pathologies and, therefore, is considered a good therapeutic target for healthy aging [17].

This aspect involves the alteration of the nutrient sensing pathways, which can be mainly regrouped into IGF (insulin-like growth factor)/insulin, TOR (target of rapamycin), and AMPK (AMP-activated protein kinase) pathways and sirtuins [17,18,19]. It has been extensively reported that the induction of nutrient sensing pathways has multiple effects, such as improvements in mitochondrial metabolism, a reduction in oxidative stress, and the modulation of protein synthesis, and, at the systemic level, has a positive impact on insulin resistance, inflammation, and vascular stiffness [17]. Understanding the molecular actors of metabolism and the presence of adjustable nutrient sensing pathways has been an important hallmark in the management of metabolic disorders such as type 2 diabetes mellitus (T2DM), metabolic syndrome, and sarcopenia. These findings clarify the properties of calorie restriction regimens and induce the design and screening of molecules able to target nutrient sensing pathways to improve quality of life in a vast segment of the population (calorie restriction mimetics), improving the quality of life of numerous individuals [20]. Analogously, the evidence that some of the modifications induced by physical exercise exploit common nutrient sensing pathways [21] makes these pathways an interesting target for the design of exercise mimetics and the use or repurposing of some calorie restriction mimetics.

**Figure 2:**
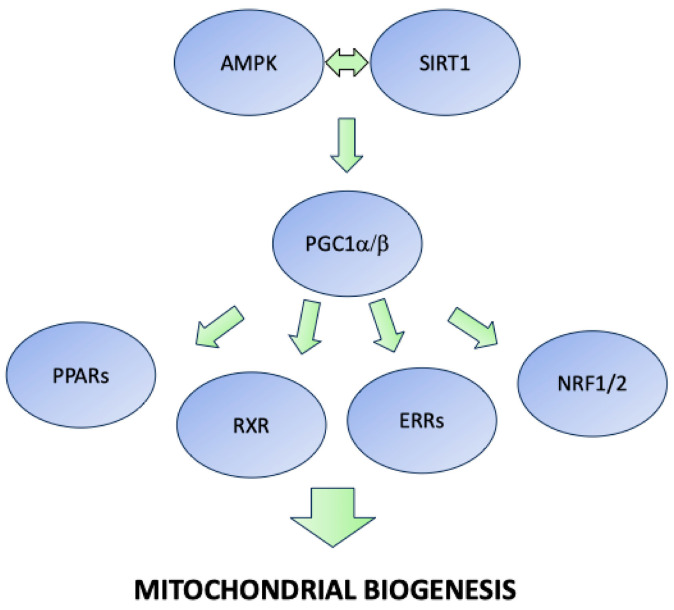
Diagrammatic representation of sirtuins and AMPK downstream interactors. Abbreviations: AMPK (AMP-activated protein kinase), SIRT (sirtuin), PGC1 (peroxisome proliferator-activated receptor γ coactivator), PPARs (peroxisome proliferator-activated receptor γ), RXR (retinoid X receptor), ERRs (estrogen-related receptors), NRF1/2 (nuclear factor E2-related factor 1/2).

The grouping of the different compounds is quite difficult for some obvious reasons, such as the presence of multilevel interactions in the nutrient sensing pathway, the central role of mitochondria in oxidative stress and aging pathways [22], and the presence of key regulators such as AMPK, PGC1s (peroxisome proliferator-activated receptor γ coactivator 1), and PPARs (peroxisome proliferator-activated receptor γ), which have several downstream targets (see Figure 2). We have grouped them into the main reported categories below.

#### 3.1.1. Sirtuins, AMPK, PGC1, and PPAR Agonists

The discovery of sirtuins and their interaction with the AMPK pathway in the regulation of mitochondrial biogenesis has opened up a research field oriented toward the finding of strategies that improve the lifespan and health span. Actually, AMPK is able to sense low ATP levels, inducing a cellular response that induces ATP production and reduces ATP consumption [23]. Accordingly, AMPK activation plays a crucial role both in the nutrient sensing pathway and in the modulation of the response to exercise [24]. As depicted in Figure 2, the modulation of this pathway involves PGC1α/β and other multiple downstream effectors that occur together with improvements in mitochondria quality.

Among the first synthesized agents able to target the AMPK pathway (Table 1), 5-amino-4-imidazolecarboxamide riboside (AICAR) is a well-characterized synthetic molecule proven to improve the metabolic condition of several cell types and organs comparable to the action of physical exercise [25]. Although it has been described as an insulin mimetic (and calorie restriction mimetic), considering that the insulin pathway is a major target of exercise, this compound has also been investigated for its properties as an exercise mimetic in several tissues and organs. AICAR had a positive action on the skeletal muscles of mice affected by spinal muscular atrophy [26] and induced phenotypic changes in the skeletal muscle of a mouse model of Huntington’s disease by interacting with PGC1α [27]. It also improves hepatic metabolism [28] and benefits brain function [29]. Interestingly, it has been reported that the addition of AICAR and free fatty acids to C2C12 cells modulates the expression of IL-6 and IL-15, providing further evidence that the pharmacological control of the skeletal muscle nutrient sensing pathway could play a fundamental role in the circulation of myokines [30].

There are also less-investigated AMPK activators, identified by the current literature search. R419 has been shown to improve exercise capacity and skeletal muscle insulin sensitivity in obese mice [31]. Interestingly, O304 has been demonstrated to reduce fasting plasma glucose levels and insulin resistance, to improve microvascular perfusion, with a reduction in blood pressure in mice and in patients with T2DM [32], and to improve not only exercise capacity but also cardiac function in aging mice [33]. Remarkably, recent research has also been focused on the design, synthesis, and testing of short-acting pan-AMPK activators, which, in contrast to long-acting activators, have a transient effect on glucose transport and no effect on glycogen accumulation, behaving similarly to a short exercise session [34,35]. Interestingly, Muise and collaborators report that the transcriptome response in the skeletal muscle, heart, liver, and white and brown adipose of lean and obese mice and rats treated with short-acting pan-AMPK activators is similar to that observed with exercise [35]. In these tissues, pan-AMPK activators interact with multiple pathways physiologically governed by AMPK, such as pathways regulating glucose and lipid metabolism, mitochondrial biogenesis, cell cycle regulation, and catabolism. These data emphasize AMPK’s crucial role in regulating metabolism not only in skeletal muscle but also in the heart, in adipose tissues, and in the liver, providing a pharmacological opportunity to exert a multi-organ action with only one active compound.

Concerning PGC-1α modulation, Kim and collaborators identified indoprofen from a screening for PGC-1α inducers and reported that in young and old mice presenting with dexamethasone-induced atrophy, indoprofen treatment activated the enzymes of oxidative metabolism and increased muscle mass. At the molecular level, indoprofen has been demonstrated to inhibit PDK1 (phosphoinositide-dependent kinase-1), activating AKT (Ak strain transforming) and AMPK, which induces PGC-1α synthesis and function [36]. Moreover, a recent paper described a PGC-1α activator, ZLN005, which has been reported to improve mitochondrial respiration through the stimulation of AMPK and mitochondrial transcription factor A (Tfam) promoter activity [37]. Due to its centrality in the nutrient sensing pathways, PGC1α signaling has been shown to be stimulated by compounds of different origin (included in other paragraphs), such as carbon monoxide in a cell-based therapy, which has been reported to improve sarcopenia in denervated mice skeletal muscles by activating Akt signaling and promoting mitochondrial biogenesis [38]. PGC1α modulators are reported in Table 1.

Regarding PPAR modulators, GW501516, which is an agonist of PPARδ, has been demonstrated to improve glucose homeostasis, increase mitochondrial activity, and attenuate body weight and fat mass accumulation in obese mice [39]. Both GW501516 and AICAR induce metabolic remodeling in the skeletal muscles of dystrophin-deficient mdx mice [40]. Possibly, these two molecules improve structural integrity and reduce the degeneration/regeneration of skeletal muscles by modulating defective oxidative metabolism. GW0742, another PPARβ/δ agonist, has been shown to induce immunometabolic effects similar to exercise in obese female mice undergoing weight loss by improving insulin sensitivity and reducing inflammation [41]. Among the PPAR modulators, Sadasivuni and collaborators reported on the CNX-013-B2 rexinoid compound, which is able to bind and activate RXRs (retinoid X receptors), and to selectively activate PPARα,β,δ,γ [42]. Its administration to obese mice improved insulin sensitivity and glucose tolerance, reduced glycemic and lipid levels, and significantly reduced body weight. Moreover, CNX-013-B2 specifically modulates the expression of those genes that are targets of RXR nuclear translocation and are involved in glucose and fat metabolism. In the liver, authors report the increase in ApoAII, ACOX1, MDR3, SREBP1c, and SCD1; in adipose tissue, PPAR γ, SREBP1c, and SCD1; and in muscle tissue, PDK4, DiO2, and UCP3 [42]. As discussed for pan-AMPK activators, CNX-013-B2 could have good potential because molecules that exert the multi-organ regulation of metabolic pathways could be more easily adapted to mimic the multi-organ effect of exercise. PPARs modulators are reported in Table 1.

The literature search provides evidence of molecules that are able to modulate the sirtuin pathway. For example, Song and collaborators treated adult male mice with MDL-801, a sirt6 deacetylation activator, showing an improvement in exercise endurance and a myofiber metabolic switch toward oxidative type by CREB-dependent Sox6 suppression [43]. Another molecule able to interact with the sirtuin pathway is nicotinamide mononucleotide, a nicotinamide adenine dinucleotide (NAD) precursor, which is a cofactor in several crucial metabolic processes. Notably, an adequate ratio of reduced and non-reduced NAD is essential for the maintenance of several metabolic pathways, among which is the activation of sirtuin deacetylases. Since aging envisages a reduction in NAD levels, it has been suggested that NAD precursors can partially reverse aging by activating sirtuin deacetylases [44]. Accordingly, nicotinamide supplementation has been demonstrated to promote vascular angiogenesis in cell culture systems, and interestingly, its administration to aging mice has been shown to restore capillary density and improve endurance in a treadmill test [45]. It has also been demonstrated to partially mimic and complement the action of exercise on the gut microbiome of obese mice [46].

The expression and function of sirtuins, AMPK, PGC1, and PPAR key regulators of metabolic pathways have been shown to be indirectly modulated by several compounds, some of which are reported in this subsection and detailed in Table 1 and others that have been grouped in other categories described below.

**Table 1:** List of sirtuins, AMPK, PGC1, and PPAR agonists. The columns report the active principle, the tissue/organ investigated, the main effects, the proposed targets, and the first author and year.

Active Principle	Tissue/Organ	Main Effects	Proposed Targets	First Author, Year
Carbon monoxide	Skeletal muscle	Improvement in skeletal muscle loss, increase in mitochondrial biogenesis factors	Metabolism, PGC-1 alpha	Noguchi, 2024 [38]
Nicotinamide mononucleotide	Gut	Restored predicted microbial functions	Metabolism	Yu, 2024 [46]
Sulforaphane, urolithin A, and ZLN005	Skeletal muscle	Improved mitochondrial respiration	Mitochondrial metabolism, AMPK, Nrf-2	Moradi, 2024 [37]
MDL-801	Skeletal muscle	Enhanced endurance performance, increased oxidative fibers and mitochondrial oxidative capacity	Mitochondrial metabolism, Sirt6	Song, 2022 [43]
O304, pan-AMPK activator	Cardiac system, systemic	Prevention of insulin resistance, improved cardiac function,	Metabolism, AMPK	Ericsson, 2021 [33]
Indoprofen	Skeletal muscle	Activation of oxidative metabolism, increased muscle mass	Metabolism, AMPK	Kim, 2020 [36]
GW0742	Lymphoid tissue, skeletal muscle, systemic	Weight loss, visceral fat mass reduction, better insulin sensitivity, reduced inflammation	Metabolism, AMPK	Garf, 2019 [41]
Small molecule activators of AMPK	Skeletal muscle, heart, liver, adipose tissue	Better glucose tolerance, improved glucose accumulation and glycogen mobilization, better fatty acid oxidation	Metabolism, AMPK	Muise, 2019 [35]
AICAR	Liver, systemic	Improved hepatic metabolism	Metabolism, AMPK	Linecker, 2020 [28]
Nicotinamide mononucleotide	Skeletal muscle, vessels	Angiogenesis promotion	Metabolism, Sirt-1	Das, 2018 [45]
AICAR	Skeletal muscle, brain	Improved muscle phenotype	Metabolism, AMPK	Paré, 2017 [27]
AICAR	Skeletal muscle, nervous tissue	Improved skeletal muscle atrophy and neuromuscular junctions, no effects on motoneuron glutamatergic synapse or on microglial and astroglial reaction	Metabolism, PGC-1 alpha	Cerveró, 2016 [26]
R419	Skeletal muscle, systemic	Improved insulin sensitivity, improved exercise capacity	Metabolism, AMPK	Marcinko, 2015 [31]
AICAR	Skeletal muscle, brain	Better synaptic plasticity, cell proliferation, gene expression, oxidative stress	Metabolism, AMPK, Myokines	Guerrieri, 2015 [29]
CNX-013-B2	Skeletal muscle, adipose tissue, liver	Improved insulin sensitivity and glucose tolerance, better body weight, alteration in gene expression	Metabolism, PPAR alpha, beta, delta	Sadasivuni, 2014 [42]
Free fatty acids, adrenaline, AICAR	Skeletal muscle	Modulation of Il-15 and Il-6 expression	Metabolism, myokines	Sánchez, 2013 [30]
AICAR, GW501516	Skeletal muscle	Influence on body weight and animal activity, increased oxidative capacity, satellite cell activation, better muscle fibrosis	Metabolism, PGC-1 alpha	Jahnke, 2012 [40]
GW501516, PF-879	Skeletal muscle, adipose tissue, liver, systemic	Changes in body weight, fat mass and lean mass, better mitochondrial activity and fiber size, better lipid profiles, improved physical activity	Metabolism, PPAR-gamma, myokines, myostatin	Bernardo, 2010 [39]
GW501516, AICAR	Skeletal muscle, systemic	Better muscle gene expression, muscle remodeling, increased running endurance	Metabolism, AMPK-alpha, PPAR-delta	Narkar, 2008 [25]

#### 3.1.2. Estrogen Receptors (ERs) and Estrogen-Related Receptor (ERR) Ligands

ERs and ERRs play crucial roles in skeletal muscle maintenance, physiology, and metabolism [47,48] and, therefore, play a crucial role in aging, maybe because they are both key regulators of mitochondrial quality (see Figure 2). ERRs, which comprise ERRα, ERRβ, and ERRγ, are nuclear receptors with sequence similarities to ERα but do not bind to endogenous estrogen, have distinct DNA response elements, and require a coactivator to exert their transcriptional activity [49].

Among the agents that exert exercise-mimetic properties via the activation of the ER- and ERR-dependent pathways, there are several products of natural origin (Table 2), some of them easily introduced with a balanced diet or with the addition of supplements. Nirmala and collaborators reported on the properties of linarin, extracted from *Chrysanthemum zawadskii*, which ameliorates sarcopenia in aging mice via the modulation of PPARδ, ERRγ, and sestrin 1 [50]. In turn, sestrin 1, a member of the sestrins family, is a metabolic regulator that activates the AMPK and mTOR/Akt pathways [51], inducing a positive loop that improves mitochondrial function and tissue proteostasis. Meng and collaborators showed that an extract of *Lycium barbarum*, or Goji berries, increases the percentage of oxidative muscle fibers and improves muscle endurance by modulating the PKA–CREB signaling pathway and activating ERRγ [52]. Seferos and collaborators reported that *Hypericum Perforatum* L. restored bone mass in swimming stressed rats [53]. Although authors do not provide evidence of the signaling pathways involved in the process, bone mass changes could be ascribed to the presence of hypericin, hyperforin, hyperoside, and flavonoids with an estrogen-mimetic effect.

Besides natural products and phytoestrogens, ERs and ERRs have also been targeted by products of synthesis (Table S1). In a recent study, Ponnusamy and collaborators showed that the pharmacologic activation of ERβ by βLGND2 increases mitochondrial function and upregulates markers of adipose tissue browning [54]. Concerning ERRs, Billon and collaborators reported the exercise-mimetic properties of a synthetic ERR agonist, SLU-PP-332. The administration of this ERR agonist to obese mice has been shown to improve exercise capacity, energy expenditure, fatty acid oxidation, and insulin sensitivity and to reduce fat mass accumulation [55,56].

**Table 2:** List of natural products with exercise-mimetic properties. The columns report natural products/compounds, the tissue/organ investigated, the main effects, the proposed targets, and the first author and year.

Natural Product/Compound	Tissue/Organ	Main Effects	Proposed Targets	First Author, Year
Eugenol	Skeletal muscle, adipose tissue	Increased exercise endurance, fiber-type switch, white fat browning, lipolysis	Metabolism, myokines, TPRV1	Huang, 2024 [57]
Eicosapentaenoic acid	Skeletal muscle, systemic	Increased oxidative metabolism, increased body fat oxidation, better muscle performance	Metabolism, PPR-delta	Komiya, 2024 [58]
Chrysanthemum zawadskii, linarin	Skeletal muscle	Prevention of sarcopenia and muscle loss, better mitochondrial function and proteostasis	Metabolism, PPR-delta, ERR-gamma	Nirmala, 2024 [50]
Sulforaphane, urolithin A, and ZLN005	Skeletal muscle	Improved mitochondrial respiration	Mitochondrial metabolism, AMPK, Nrf-2	Moradi, 2024 [37]
Resveratrol	Vessels	Prevention of endothelial dysfunction	Oxidative stress, SIRT-1	Kim, 2023 [59]
Zynamite(^®^), quercetin	Skeletal muscle	Enhanced physical performance	GSK3beta, stress kinases	Martinez-Canton, 2023 [60]
Essential amino acids	Brain, primary cortical neurons	Improved mitochondrial biogenesis, antioxidant response	Mitochondrial metabolism, eNOS/mTOR	Ragni, 2023 [61]
7,8-DHF@ZIF-8, 7,8-Dihydroxyflavone	Bone, vessels	Improved osteogenesis and angiogenesis	BDNF	Sun, 2023 [62]
Limonium tetragonum	Skeletal muscle	Enhanced exercise endurance, increased oxidative fibers, increased mitochondrial content	Mitochondrial metabolism, PKA–CREB–PGC1 alpha	Lee, 2022 [63]
(-)-Epicatechin	Skeletal muscle	Increased fiber size	MyomiRs	Palma-Flores, 2023 [64]
Multi-ingredient supplement	Skin	Upregulation of proteins involved in mitochondrial function and oxidative phosphorylation, improvement in antioxidant activity	Oxidative stress, PPAR-gamma, Il-15	Rebalka, 2022 [65]
d-Allulose	Skeletal muscle, systemic	Improved performance, better insulin sensivity	Metabolism, AMPK, PGC-1 alpha	Liu, 2022 [66]
Trehalose	Brain	Improved learning and memory	AMPK, TOR, autophagy	Pan, 2022 [67]
Epicatechin	Central nervous system, skeletal muscle	Resilience to depression	Kynurenine aminotransferases, PGC-1 alpha-PPAR-delta/alpha	Martínez-Damas, 2021 [68]
Olive oil	Skeletal muscle	Improved running endurance, increased muscle triacylglycerol	Metabolism, DGAT1	Komiya, 2021 [69]
Resveratrol	Brain, skeletal muscle	Better capillary density in the ipsilesional hemisphere, mitigation of stroke-induced muscle fiber changes	Sirtuins	McDonald, 2021 [70]
Lycium barbarum extract	Skeletal muscle	Increase in muscle mass and endurance, switch from glycolytic to oxidative metabolism	Metabolism, ERR-gamma, sirtuins, PGC-1 alpha/beta	Meng, 2020 [52]
cis-Banglene	Skeletal muscle	Improved glucose uptake, improve mitochondrial biogenesis	Myokines, metabolisms, IL-6, AMPK	Norikura, 2020 [71]
Epicatechin	Skeletal muscle	Modulation of skeletal muscle protein expression, better mitochondrial morphology	Regeneration	McDonald, 2021 [72]
Estradiol, resveratrol	Vessels	Enhanced basal endothelial function	Estrogen receptors	Ozemek, 2020 [73]
Ursolic acid	Skeletal muscle, bone	Improved muscle mass and bone density	No suggestion	Kang, 2019 [74]
Multi-ingredient supplement	Locomotor system	Improved mean survivorship, improved morphological properties, improved jumping	No suggestion	Tran, 2018 [75]
Ursolic acid	Skeletal muscle	Improvement in atrophied muscle mass, reduction in atrophic genes expression	Atrophy, Murf-1, Atrogin-1	Kim, 2018 [76]
7,8-dihydroxyflavone (BDNF-mimetic)	Brain	Improved brain plasticity, associative learning	BDNF	Parrini, 2017 [77]
Resveratrol, metformin	Skeletal muscle	Better skeletal musle morphology and neuromuscular junction structure	No suggestions	Stockinger, 2017 [78]
Cocoa procyanidins	Skeletal muscle	Improved glucose uptake and glycogen synthesis	Metabolism, AKT	Bowser, 2017 [79]
Hypericum perforatum L.	Bone, systemic	Better testosterone levels, better bone specific weight and mass density	No suggestions	Seferos, 2016 [53]
Fenugreek	Skeletal muscle	Increased total creatine, modulation of protein expression	Metabolism, insulin	Tomcik, 2017 [80]
Linoleic acid	Skeletal muscle	Body weight reduction, better voluntary movement, better mitochondrial biogenesis	Metabolism, AMPK-alpha, PPAR-gamma	Kim, 2016 [81]
Dihydromyricetin	Skeletal muscle, systemic	Higher irisin levels	Myokines, PGC1-alpha	Zhou, 2015 [82]
Resveratrol	Lung endothelium	Attenuation of oxidative damage, better endothelial permeability and lung histomorphology	Oxidative stress, Nfr-2	Dong, 2015 [83]
Resveratrol	Skeletal muscle	No effect	Metabolism	Olesen, 2014 [84]
Ginsenoside Rg3	Cardiac system	improved cardiac adaptations and mitochondrial homeostasis	Metabolism, PGC-1alpha, Nrf-2	Sun, 2013 [85]
Resveratrol	Skeletal muscle, systemic	Variation in protein expression, better energy expenditure	Metabolism, Sirt-1	Goh, 2014 [86]
Chitooligosaccharide	Skeletal muscle	Increased mitochondrial content, improved exercise endurance	Metabolism, AMPK, PGC-1 alpha, Sirt1	Jeong, 2012 [87]
(-)-Epicatechin	Skeletal muscle, cardiac tissue	Better physical performance, regulation of oxidative phosphorylation complexes, improved mitochondrial quantity and morphology	Metabolism, oxidative stress	Nogueira, 2011 [88]
Resveratrol	Skeletal muscle, adipose tissue, bone, cardiovascular system, systemic	Prevention of muscle atrophy and loss of function, oxidative capacity maintenance and improved oxidative stress, prevention of bone demineralization	Metabolism, PGC-1 alpha, Sirt1	Momken, 2011 [89]
Cordyceps sinensis	Skeletal muscle, systemic	Improvement in endurance capacity, better glucose transport, better angiogenic and antioxidant response	Metabolism, AMPK, PGC-1 alpha	Kumar, 2011 [90]
Trichopus zeylanicus	Skeletal muscle, systemic	Anti-fatigue effect	No suggestions	Tharakan, 2006 [91]

#### 3.1.3. Antioxidants

Muscle contraction and high-intensity or prolonged exercise stimulate the production of reactive oxygen/nitrogen species (ROS/RNS), which can affect cell equilibrium and performance [92]. Moreover, the exercise stimulus is essential for the upregulation of endogenous antioxidant defenses [93] to the point that mild exercise is advised to decrease oxidative stress in older people. In the tissues, improvements in oxidative stress can be achieved in several ways, comprising the presence of molecules that directly buffer ROS and RNS, enzymes that convert ROS species such as super oxide dismutases (SODs), or the scavenging of ROS and RNS by increasing blood circulation (Table S1).

SODs are endogenous antioxidants that regulate the levels of superoxide anions to control cellular oxidative stress, and this literature search found evidence of the exercise-mimetic properties of several compounds. In this context, the positive effects of Tempol, a SOD-mimetic agent, have been reported for several tissues. Tempol has been shown to upregulate nitric oxide synthase in the kidneys of hypertensive rats [94]. It has been shown to be beneficial in a model of ischemic muscle, where it modulates the pressor response [95], possibly by hindering the effects of a reduced blood supply. Moreover, Tempol has been shown to improve central command dysfunction in a rat model of cardiac infarction [96]. Nevertheless, according to McCord and collaborators, in decerebrated rats with femoral artery occlusion, Tempol attenuated the exercise pressor reflex independently of ROS reduction [97], opening up the possibility of the presence of other mechanisms.

Brestoff and collaborators showed that the administration of manganese [III] tetrakis [5,10,15,20]-benzoic acid porphyrin, a cell-permeable SOD mimetic, to mice fed on a high-fat diet was able to ameliorate pre-existing obesity, reduce body weight gain and adipose tissue, and improve insulin action through the increase in both PKB (protein kinase B) levels and phosphorylation [98]. These data suggest that the action of manganese [III] tetrakis [5,10,15,20]-benzoic acid porphyrin exploits nutrient sensing pathways.

Moreover, the chronic systemic administration of another synthetic SOD mimetic, EUK-189, has been shown to prevent heat stress-induced liver injury by decreasing oxidative damage in aging rats [99]. Treatment with EUK-189 improves the redox status and attenuates the response to heat stress in old rats, which present better lipid peroxidation and a better histological profile of the liver.

In the group of antioxidants, it is worth mentioning trimetazidine, an inhibitor of 3-ketoacyl Co-A thiolase, which reduces fatty acid oxidation-shifting ATP production from fatty acid oxidation to glucose oxidation. Molinari and collaborators reported that trimetazidine had a positive effect on the skeletal muscles of cachectic mice with C-26 colon carcinoma, resulting in improved grip strength, an increased cross-sectional area, augmented mitochondrial biogenesis and oxidative metabolism, reduced blood glucose, and the promotion of angiogenesis [100].

As mentioned above, the oxidative equilibrium in tissue can also be improved by modulating its blood circulation. Actually, vascular dysfunction is one of the age-related problems that coincides with the increased risk of developing cardiovascular diseases. In this context, the administration of 3,3-dimethyl-1-butanol to aging mice has been demonstrated to attenuate aortic stiffness and improve endothelial function by improving the levels of oxidative stress in several experimental models. This effect has been ascribed to the inhibition of plasma levels of trimethylamine-N-oxide, a molecule produced by the gut microbiome and responsible for the increase in age-related vascular oxidative stress [101,102].

Oxidative stress reduction has been reported also for cobalt chloride, which behaves as a hypoxia mimetic, stabilizing hypoxia-inducible factor-1 (HIF-1) [103,104] and, therefore, modulating a plethora of responses that occur together in the regulation of oxygen homeostasis [105]. Preconditioning with cobalt chloride exerts several effects on skeletal muscle, such as an increase in physical performance, improved cellular oxygen sensing, an improved GSH/GSSG ratio, lipid peroxidation, and better mitochondrial biogenesis [103,104].

Worth mentioning in this section is the work of Ragni and collaborators, which reported that the administration of a balanced formula of amino acids to middle-aged mice affected by brain ischemia, similar to physical exercise, improved the mitochondrial biogenesis and antioxidant milieu and protected them from ischemic insult by interacting with the eNOS/mTOR pathway [61].

In addition to the above-reported compounds, food and medicinal plants are a source of antioxidants [106]. These will be described in the following paragraph.

#### 3.1.4. Products of Natural Origin

In the group of agents that regulate metabolism fall several plant-derived molecules and products often used in traditional medicine practices that can be introduced with diet [107] (see Table 2). Interestingly, the use of functional foods, supplements, nutraceuticals, or small bioactive ingredients as exercise mimetics seems to have good potential because they can promote a longer lifespan and a better health span by exerting numerous actions [108]. This is the case of plants that contain a mixture of antioxidants, such as polyphenols or flavonoids with estrogen-mimetic properties, and other active agents.

One of the most investigated products of natural origin is resveratrol, a pleiotropic molecule that was first defined as an antioxidant molecule, then included among the calorie restriction mimetics, and is now also categorized as an exercise mimetic. It has been described for its capability to prevent age-associated deterioration at different levels by modulating the nutrient sensing pathway, with positive consequences for skeletal muscle and systemic metabolism [109,110]. Despite the presence in the literature of some controversial data [84,111], resveratrol exerts a beneficial action on skeletal muscle, which results in a better mitochondrial metabolism, improved capillary density [112], reduced inflammatory condition [113], better physical performance, and overall improved muscle health [111,114]. Moreover, although the mechanisms are not completely elucidated, resveratrol slows the aging of the neuromuscular junction in aging mice [78]. Interestingly, this literature search found evidence that resveratrol can act as an exercise mimetic through the modulation of the metabolic pathways in several organs. It has a beneficial action, similar to exercise, on the skeletal muscle of patients with T2DM [86] and of rats with suspended hindlimbs [89] thanks to its interaction with the PGC1α and Sirt-1 pathways. Interestingly, resveratrol modulates endothelial cell health exploiting several pathways. It prevents endothelial senescence by improving oxidative stress [59], restores endothelial function in estrogen-deficient postmenopausal women [73], protects the lung–endothelial barrier through improvements in oxidative stress and the Nrf-2 (nuclear factor E2-related factor 2) pathway [83], and improves capillary density in the brain after stroke [70].

Another example of a natural antioxidant with exercise-mimetic properties is epicatechin, a natural polyphenol found in tea and cocoa [115], which has been proposed as an insulin-mimetic molecule. The administration of epicatechin induces a better physical performance, the regulation of oxidative phosphorylation complexes, and improved mitochondrial quantity and morphology in aging mice [88]. Interestingly, although the mechanisms have not been fully elucidated, epicatechin has been reported to induce mitochondrial biogenesis and the expression of markers for muscle regeneration in a clinical trial involving patients with Becker dystrophy [72]. The interaction of epicatechin with the metabolic pathways regulated by PGC1α and PPARδ/α improved resilience to chronic mild stress in a murine model of depression, indicating that it is able to interact also at the level of the central nervous system. This effect has been explained by the induction of the expression of kynurenine aminotransferase in skeletal muscles, which protects against stress-induced depression by converting kynurenine into kynurenic acid, caused by an increase in PGC1α, PPARα, and PPARδ [68]. Moreover, epicatechin regulates the expression of myomiRs, modulating the fiber size response to exercise [64]. Analogously, high molecular weight procyanidins from cocoa have been reported to improve glucose uptake and glycogen synthesis in human primary skeletal muscle cells via AKT phosphorylation [79].

Martinez-Canton and collaborators tested the antioxidant and exercise-mimetic properties of Zynamite^®^ [60], an extract from mango leaves rich in mangiferin [116] combined with quercetin, reporting an increase in Ca^2+^/calmodulin-dependent protein kinase II (CaMKII), the inhibition of GSK3beta (glycogen synthase kinase-3 beta), and a lack of NRF-2 phosphorylation, inducing the increase in NRF-2 resting levels in non-exercised muscles. Moreover, the same treatment in exercised muscles partly abrogated the stress kinase responses by phosphorylating CaMKII and GSK3beta, with the consequent modulation of NRF-2 and improvement in oxidative stress conditions [60].

The list of natural products with exercise-mimetic properties is very long. A mixture of conjugated isomers of linoleic acid has been shown to influence mitochondrial biogenesis signaling in the muscle of an obese mouse model by activating AMPKα and improving several mitochondrial markers, such as PPARδ [81]. The administration of ginsenoside Rg3, one of the active ingredients of ginseng, to sedentary rats, has been shown to improve the mitochondrial quality of heart tissue by activating the AMPK, PGC1α, and Nrf2 pathways and increasing the mRNA levels of Nrf1 (nuclear-related factor 1) and Tfam, improving endogenous antioxidant levels until similar to those in exercised rats [85]. Lee and collaborators showed that *Limonium tetragonum* improves mitochondrial biogenesis in skeletal muscle, increasing the running endurance in mice via the PKA–CREB–PGC1δ pathway [63]. *Trichopus zeylanicus* is an example of an Indian medicinal plant used in Kani tribal practices in India and by people that live at high altitudes to rapidly obtain energy against fatigue. Its administration has been reported to combat fatigue in young rats, in aged mice, and in mutant Ames dwarf mice [91]. Although the mechanism of action has not been elucidated, it has been reported that *Trichopus zeylanicus* exerts its anti-fatigue action not as an amphetamine mimetic but by exploiting other pathways [91]. The activation of metabolic responses similar to exercise have also been demonstrated for *Cordyceps sinensis*, a fungal traditional medicine used in Tibet and Nepal. Its supplementation to rats improved endurance capacity and increased the expression of several proteins, such as AMPK, PGC-1α, PPAR-δ, VEGF, GLUT-4, Nrf-2, SOD, and lactate transporters, which can be associated with better glucose transport and a better angiogenic and antioxidant response [90]. Improvements in insulin metabolism in L6C11 muscle cells has also been reported for fenugreek by Tomcik and collaborators [80].

Other compounds extracted from plants have been reported to exert exercise-mimetic activity. This is the case for sulforaphane, found in cruciferous vegetables, and urolithin A, a gut microbial metabolite, which increased the nuclear localization of Nrf-2 and the expression of antioxidant enzymes as well as the expression of AMPK and mitophagy markers [37]. This literature search also highlighted ursolic acid, a natural triterpene found in fruits and vegetables, suggested for its anti-inflammatory, antioxidant, and anticarcinogenic properties. Kim and collaborators showed that treatment with ursolic acid and low-intensity treadmill exercise in a model of hindlimb atrophy in rats was able to improve skeletal muscle atrophy [76] and bone microstructure [74]. Lastly, witnessing the collaboration between metabolic pathways and myokines, the natural flavonoid dihydromyricetin has been shown to behave as an exercise mimetic by inducing irisin secretion partially via the PGC-1α pathway [82], and cis-Banglene has been demonstrated to activate AMPK, promote glucose uptake, induce mitochondrial biogenesis, and increase the expression and secretion of IL-6 [71].

It is also worth reporting the properties of some sugar-related molecules. Trehalose, a natural disaccharide, has been demonstrated to activate autophagy and delay brain aging in aged mice by increasing AMPK phosphorylation to a comparable degree with exercise [67]. d-Allulose has been shown to improve performance and insulin sensitivity in mice [66]. Chitooligosaccharide, a glucosamine polymer derived from enzyme-digested chitosan, increased the mitochondrial content in skeletal muscle and enhanced exercise endurance in rats, which is mainly ascribable to the activation of Sirt1 and AMPK [87].

In line with the evidence that monounsaturated fatty acids (MUFAs) and polyunsaturated acids (PUFAs) may be agonists for PPARs, this literature search identified two articles reporting the potential of olive oil, rich in MUFAs [69], and fish oil, rich in PUFAs [58]. MUFAs and PUFAs have been demonstrated to exert several healthy actions, among which is a reduction in the risk of developing cardiovascular diseases and diabetes [117]. Actually, it is well demonstrated that MUFAs and PUFAs are able to reverse the cytotoxic action of saturated fatty acids induced by inadequate diet or by obesity [118,119,120].

Interestingly, the introduction of olive oil into the diet of mice has been shown to improve running endurance and increase the intramuscular content in triacylglycerol via the upregulation of diacylglycerol O-acyltransferase1 [69]. Moreover, as reported by Komiya and collaborators, fish oil has potential as an exercise mimetic since its administration induces a fiber-type transition toward a slower phenotype in rats’ muscles through the activation of the PPARδ and AMPK pathways by eicosapentaenoic acid [58].

Finally, in this group fall some of the products of natural origin that have been already described in Section 3.1.2 because they are involved in the ER- and ERR-mediated response and some that interact with the myokine pathways, which will be described in the following paragraphs (see Table 2 and Table 3).

#### 3.1.5. Products with Miscellaneous Targets

In this search, we found several products whose mechanisms of action are poorly defined or that are able to modulate mechanisms that cannot be grouped in one of the above-mentioned categories.

In this section falls a selection of endogenous molecules that have a physiological role in the maintenance of the metabolic equilibrium of an individual, and therefore, they could be exploited as exercise mimetics. An example is provided by incretins, a group of gut peptides that are secreted in a nutrient- and glucose-dependent way to stimulate insulin secretion and glucose uptake [121]. Accordingly, the incretin mimetic exenatide has shown potential as an exercise mimetic because it is able to restore the insulin secretory pattern, reduce glycosylated hemoglobin, improve fasting plasma glucose, and reduce body weight in patients with T2DM [122,123,124]. Moreover, treatment with semaglutide, another incretin mimetic, improves glucose metabolism and reduces body weight in patients with obesity affected by polycystic ovary syndrome [125].

Interestingly, Lee and collaborators report that the chronic administration of the adiponectin mimetic AdipoRon in diabetic mice restored the neuroplasticity in the hippocampus of these mice [126]. This is ascribed to the physiological activity of adiponectin, which, secreted by adipocytes, exerts both central and metabolic positive effects [127]. Actually, Lee and collaborators showed that AdipoRon increased neuronal proliferation and differentiation via the activation of AMPK and PGC-1α and increased brain-derived neurotrophic factor (BDNF) levels in the hippocampus [126].

Another example is provided by glucagon-like peptide 1 (GLP-1) mimetics, which are used in the treatment of type 2 diabetes and have been described for their neuroprotective properties in models for degenerative disorders. Zhang and collaborators reported that the GLP-1 analogue (Val8)GLP-1-glu-PAL reduced motor impairment, dopamine production, and pro-apoptotic signaling in substantia nigra [128]. Analogously, the same working group reported that oxyntomodulin, which is exploited in the treatment of diabetes by activating the GLP-1 and glucagon receptor, displayed a neuroprotective effect in a mouse model of Parkinson’s disease [129].

A further case of exploitation of endogenous molecules is provided by MOTS-c, which is a 16-amino acid hormone encoded by mitochondrial rRNA, whose plasma levels have been shown to be correlated with insulin resistance [130]; its administration to obese mice promoted a better metabolic homeostasis [130,131].

Dong and collaborators reported on a human trial entailing the administration of PF-05231023, a FGF21 receptor complex agonist, described for its potential to reduce blood glucose, lipid levels, and body weight [132]. Although FGF21 receptor effects are still not completely elucidated, its activation with PF-05231023 decreased triglyceride, total cholesterol, and low-density lipoprotein cholesterol in patients with T2DM [133].

Finally, lactate administration to mice has been demonstrated to improve weight, glucose, and insulin levels and the expression of mitochondrial genes and to induce specific exercise-related changes to the brain and liver [134].

This section, although it can be considered a borderline case, also includes agents that have a central action in inducing physical activity. This is the case of GABAergic mimetics, such as sodium valproate and phenibut, which have been demonstrated to reduce immobility in stressed rats and mice [135,136].

### 3.2. Myokines

In response to exercise, skeletal muscle tissue produces myokines, which establish crosstalk between the muscle and other organs as well as within itself, exerting a beneficial multi-organ action [12]. Moreover, the presence of crosstalk between myokines and nutrient sensing pathways [16] implies that agents targeting myokines, directly or indirectly, also interact with the above-mentioned pathways, possibly exerting a wide range of effects. This evidence makes myokines interesting targets for modulating the plasticity of both muscle and other organs in conditions where exercise protocols are not tolerable. The present literature search found evidence of two major targets, irisin and BDNF.

Irisin itself has been shown to behave as an exercise mimetic in several experimental models by positively impacting several tissues. In the context of the self-action of myokines, irisin has been reported to induce some of the effects induced by exercise. As shown by Momenzadeh and collaborators, the administration of irisin to male mice induced some of the molecular modifications produced either from resistance or endurance training [137]. Moreover, irisin has been shown to prevent dexamethasone-induced atrophy in C2C12 myotubes [138]. Interestingly, this literature search found evidence of an important role for irisin in the skeleton and cartilage. Irisin has been shown to enhance osteoblast differentiation [139], to prevent disuse-induced osteocyte apoptosis [140], and to increase cell proliferation and matrix deposition in three-dimensional cultures of human articular chondrocytes [141]. Not less important, irisin has been shown to reduce sexual dysfunction induced by a high-fat diet in male rats [142]. As mentioned above, to highlight the interplay between metabolic balance in muscle and the production of myokines, irisin secretion can be induced by dihydromyricetin, which stimulates its secretion partially via the PGC-1α pathway [82].

The present systematic search also found evidence of the potential to target BDNF, which is the most studied member of the neurotrophins family. It was initially known for its secretion and action in the central nervous system [143] and was then demonstrated to be released during exercise [144] and also produced by the skeletal muscle tissue itself [145]. Interestingly, BDNF has been confirmed to be produced by skeletal muscle cells in response to contraction and to induce fat oxidation via the activation of AMPK, with a strict relationship between myokine production and metabolism modulation observed [146]. In agreement with data that suggest a role for BDNF in osteogenesis, 7,8-Dihihydroxyflavone, a plant-derived flavonoid, has been reported to promote the modulation of genes that are involved both in osteogenic and angiogenic pathways in MC3T3-E1 and HUVEC cell cultures [62]. Moreover, this same compound administered to a mouse model of Down syndrome has been shown to rescue synaptic plasticity, improving both learning and memory [77].

Myokine activation can also be induced by natural compounds or special diets. As mentioned above, cis-Banglene, a *Zingiber purpureum*-derived bioactive molecule, has been demonstrated to activate AMPK, promote glucose uptake, induce mitochondrial biogenesis, and increase the expression and secretion of interleukin (IL)-6 [71]. Another example is provided by eugenol, a compound with multiple actions, found in various plants and spices, which has been demonstrated to interact with the transient receptor potential vanilloid 1 (TRPV1) [147]. Its administration to mice increased IL-15 expression, improved endurance capacity, promoted the switch of fast-to-slow muscle fibers, and induced fat browning and lipolysis [57]. Moreover, authors demonstrated that, in C2C12 cells, eugenol modulates IL-15 levels by inducing the TRPV1-mediated CaN/NFATc1 signaling pathway [57]. Finally, Rebalka and collaborators reported that a multi-ingredient supplement (containing coenzyme Q10, alpha lipoic acid, resveratrol, curcumin, zinc, lutein, astaxanthin, copper, biotin, and vitamins C, D, and E) was able to improve mitochondrial metabolism in skin fibroblasts via the induction of PPARγ through IL-15 signaling [65].

**Table 3:** List of exercise mimetics that exploit myokine pathways. The columns report the active principle, the tissue/organ investigated, the main effects, the proposed targets, and the first author and year.

Therapeutic Agent	Tissue/Organ	Main Effects	Proposed Targets	First Author, Year
Eugenol	Skeletal muscle, adipose tissue	Increased exercise endurance, fiber-type switch, white fat browning, lipolysis	Metabolism, myokines, TPRV1	Huang, 2024 [57]
Irisin	Cartilage, bone	Improved extracellular matrix synthesis, improved chondrogenic differentiation	ERK phosphorylation, irisin	Posa, 2023 [141]
7,8-DHF@ZIF-8, 7,8-Dihydroxyflavone	Bone, vessels	Improved osteogenesis and angiogenesis	BDNF	Sun, 2023 [62]
Multi-ingredient supplement	Skin	Upregulation of proteins involved in mitochondrial function and oxidative phosphorylation, improvement in antioxidant activity	Oxidative stress, PPAR-gamma, Il-15	Rebalka, 2022 [65]
Irisin	Reproductive organs	Better sexual performance, improved sperm morphology and motility, reduced testicular damage	Myokines, irisin	Yardimci, 2022 [142]
Irisin	Skeletal muscle	Differential expression of muscle proteins	Myokines	Momenzadeh, 2021 [137]
cis-Banglene	Skeletal muscle	Improved glucose uptake, improved mitochondrial biogenesis	Myokines, metabolisms, IL-6, AMPK	Norikura, 2020 [71]
Irisin	Skeletal muscle	Attenuation of dexamethasone-induced atrophy	Myokines, irisin	Chang, 2020 [138]
Irisin	Bone	Inhibition of apoptosis	Myokines, apoptosis, Erk1/Erk2, caspase 9/3	Storlino, 2020 [140]
7,8-dihydroxyflavone	Brain	Improved brain plasticity, associative learning	BDNF	Parrini, 2017 [77]
Dihydromyricetin	Skeletal muscle, systemic	Higher irisin levels	Myokines, PGC1-alpha	Zhou, 2015 [82]
Irisin	Bone	Enhanced differentiation	Myokines	Colaianni, 2014 [139]

### 3.3. Physical Approaches

In recent years, the application of whole-body vibration (WBV) has emerged as an exercise mimetic that can be applied to those individuals that cannot perform conventional exercise protocols because of physical problems such as aging, frailty, or neuromuscular diseases. During WBV protocols, the subject stands on a platform that produces sinusoidal oscillations that result in vibrations transmitted to the subject through the legs, producing accelerations of different intensities [148]. The search found five articles reporting the effects of WBV under different conditions. WBV has been shown to mimic the effects of exercise in male obese mice, improving metabolism and the overall condition of several organs and tissues. WBV-treated mice display reduced muscle atrophy, better glycemic control and insulin sensitivity, and reduced hepatic steatosis [149]. Moreover, as reported by Yu and collaborators, WBV improved the polarization of omental macrophages and changed the fecal microbiome of obese mice [150]. WBV has also been demonstrated to be beneficial for several tissues in human trials. The application of WBV protocols in healthy subjects has been demonstrated to induce important changes in glucose metabolism by reducing blood glucose levels and insulin resistance and to positively affect skeletal muscle tissue by improving muscle oxygenation [151]. Moreover, to a lesser extent, compared to knee extension exercises, WBV has been shown to increase microvascular blood flow in skeletal muscle [152]. Interestingly, similar to exercise bouts, WBV induces the production of IL-6. The induction of myokine secretion by WBV has also been demonstrated in individuals who are obese or overweight, who, after a WBV protocol, displayed higher blood concentrations of decorin and myostatin [153].

Another physical approach that has emerged as a potential exercise mimetic is heat stress [154], which is suggested to induce modifications similar to exercise. However, the application of these methods is still considered debatable. Passive heating in patients with T2DM has been shown to improve energy expenditure [155], and far-infrared therapy in mice has been shown to promote endurance and glucose metabolism, modulate microbiota homeostasis, and activate AMPK [156]. However, Hussain and collaborators performed an analysis of the physiological response to infrared sauna versus exercise in healthy women and found that blood pressure, arterial stiffness, and heart rate variability responses were similar, while sweating and the tympanic and back skin-surface temperature were differently affected by the two protocols [157]. Interestingly, contrary to exercise, infrared sauna did not increase breathing rates [157]. Therefore, the authors suggest a careful approach to thermal strategies because they could have different effects.

## 4. Conclusions

In an attempt to provide a schematic view of exercise-mimetic therapeutic agents, we divided exercise mimetics into three main groups, those that regulate metabolic pathways, those that participate in myokine pathways, and mechanical/physical approaches. In light of the reported observations, we think that a rigid classification of exercise-mimetic compounds is not possible due to the pleiotropic effect of several substances, the multiple consequences of exercise, and most importantly, because of the crosstalk among signaling pathways that regulate the body response to exercise. Possibly, a single bioactive molecule would not be adequate for simulating the effects of physical activity because of its broad-ranging consequences in different tissues and organs. In this context, it could be hypothesized that the promising effects of plant extracts or multi-ingredient compounds [65,75] could be the result of the presence of multiple bioactive substances that target diverse pathways.

Interestingly, this review further stresses the strict relationship between diet and exercise, not only in their molecular pathways and effects on the body but also when searching for strategies for health promotion, such as the screening and investigation of new compounds able to improve the quality of aging. Actually, a good number of products of natural origin, which are assimilable with diet (examples are phytoestrogens, resveratrol, epicatechin, olive oil, and others), have been demonstrated to behave as exercise mimetics. Among these, some have been shown to interact with the nutrient sensing pathway or with insulin metabolism, which are targets of nutritional strategies. Assuming that the physiological crosstalk between metabolism and diet and metabolism and physical exercise should not be considered as separate entities, it could be helpful to reconsider from a wider perspective some known compounds, such as calorie restriction mimetics or insulin mimetics, and verify their exercise-mimetic potential.

Although skeletal muscle tissue is one of the principal actors in metabolism regulation and can be modulated by exercise (and also diet), several articles from the literature search highlight the importance of the impact of exercise and exercise mimetics on other organs, such as the liver, brain, bone, and the cardiovascular and gastrointestinal systems (see Table S1), further emphasizing the interplay among organs. This can be a further reason to find and apply nutritional and physical exercise strategies from a wider perspective, where experts in several fields could benefit from each other’s information and knowledge to implement healthy aging.

A limitation of the present review resides in the fact that some therapeutic agents have multiple targets, which are differently associable with exercise effects. This can be the case for antidepressants, which induce a central-dependent increase in activity. Moreover, it cannot be excluded that some agents have been missed in this literature search because they are not classified in the exercise-mimetic therapeutic category, they were developed before the introduction of the concept of exercise mimetics, or they have non-realistic applications.

Undoubtedly, as witnessed by the relatively low proportion of research articles compared to reviews, further work to discover new and fully elucidate the potential of existing exercise mimetics in the daily combat against aging is still necessary.

## Data Availability

No new data were created or analyzed in this study.

## Supplementary Materials

The following supporting information can be downloaded at: https://www.mdpi.com/article/10.3390/nu17060969/s1, Table S1: List of the selected articles reporting first Author and year, therapeutical agent, experimental model, tissue/organ investigated, main effects, and proposed targets.
