# Supplementary material for: Exercise Mimetics in Aging: Suggestions from a Systematic Review
Source: Nutrients. 2025 Mar 10;17(6):969. doi: 10.3390/nu17060969 (PMC11944853; doi:10.3390/nu17060969)
Supplement: Supplementary file 1 [file nutrients-17-00969-s001.zip › nutrients-3508846-supplementary.pdf]

Table S1. List of the selected articles reporting first Author and year, therapeutical agent, experimental model, tissue/organ investigated, main effects, and proposed targets.

First Author, Year	Title	Therapeutical Agent	Experimental Model	Tissue/organ	Main effects	Proposed targets
Broniec MN	The decorin and myostatin response to acute whole body vibration: impact of adiposity, sex, and race	Whole body vibration	Human trial, obese, overweight and normal weight adults	Systemic	Systemic release of decorin and myostatin	Myokines
Li, 2024	Far-infrared therapy promotes exercise capacity and glucose metabolism in mice by modulating microbiota homeostasis and activating AMPK	Far-infrared hyperthermia	Mice	Skeletal muscle, gut	Increased exercise endurance, altered gut microbiome, enhanced glucose uptake	Glucose metabolism, AMPK
Huang, 2024	Eugenol mimics exercise to promote skeletal muscle fiber remodeling and myokine IL-15 expression by activating TRPV1 channel	Eugenol (natural compound)	Mice, C2C12 cells	Skeletal muscle, adipose tissue	Increased exercise endurance, fiber type switch, white fat browning, lipolysis	Metabolism, Myokines, TPRV1
Komiya, 2024	Eicosapentaenoic acid increases proportion of type 1 muscle fibers through PPARδ and AMPK pathways in rats	Eicosapentaenoic acid	Rats, L6 cells	Skeletal muscle, systemic	Increased oxidative metabolism, increased body fat oxidation, better muscle performance	Metabolism, PPR-delta
Nirmala, 2024	Exercise-induced signaling activation by Chrysanthemum zawadskii and its active compound, linarin, ameliorates age-related sarcopenia through Sestrin 1 regulation	Chrysanthemum zawadskii, Linarin	Aged mice	Skeletal muscle	Prevention of sarcopenia and muscle loss, better mitochondrial function and proteostasis	Metabolism, PPR-delta, ERR-gamma
Noguchi, 2024	Carbon monoxide-loaded cell therapy as an exercise mimetic for sarcopenia treatment	Carbon monoxide	Mice, C2C12 cells	Skeletal muscle	Improvement of skeletal muscle loss, increase of mitochondrial biogenesis factors	Metabolism, PGC-1alpha
Yu, 2024	Complementary yet divergent effects of exercise and an exercise mimetic on microbiome in high-fat diet-induced obesity	nicotinamide mononucleotide	Obese mice	Gut	Restored predicted microbial functions	Metabolism
Billon, 2024	A Synthetic ERR Agonist Alleviates Metabolic Syndrome	SLU-PP-332, agonist for the	Obese mice	Skeletal muscle	Improved fatty acid and glucose metabolisms,	Metabolism, ERRs

		estrogen-related receptor			improved energy expenditure	
Moradi, 2024	Sulforaphane, Urolithin A, and ZLN005 induce time-dependent alterations in antioxidant capacity, mitophagy, and mitochondrial biogenesis in muscle cells	Sulforaphane, Urolithin A, and ZLN005	C2C12 cells	Skeletal muscle	Improved mitochondrial respiration	Mitochondrial metabolism, AMPK, Nrf-2
Kim, 2023	Aerobic Exercise-Mimetic Effects of Resveratrol on the Prevention of Vascular Endothelial Senescence	Resveratrol	HUVEC cells	Vessels	Prevention of endothelial dysfunction	Oxidative stress, SIRT-1
Martinez-Canton, 2023	A Mango Leaf Extract (Zynamite(®)) Combined with Quercetin Has Exercise-Mimetic Properties in Human Skeletal Muscle	Zynamite(®), Quercetin	Human	Skeletal muscle	Enhanced physical performance	GSK3beta, Stress kinases
Ragni, 2023	A balanced formula of essential amino acids promotes brain mitochondrial biogenesis and protects neurons from ischemic insult	essential amino acids	Middle aged mice	Brain, primary cortical neurons	Improved mitochondrial biogenesis, antioxidant response	Mitochondrial metabolism, eNOS/mTOR
Billon, 2023	Synthetic ERRα/β/γ Agonist Induces an ERRα-Dependent Acute Aerobic Exercise Response and Enhances Exercise Capacity	SLU-PP-332, agonist for the estrogen-related receptor	Mice, ERRalpha knockout mice, C2C12	Skeletal muscle	Enhanced exercise endurance, enhanced mitochondrial respiration	ERRs
Posa, 2023	Irisin Role in Chondrocyte 3D Culture Differentiation and Its Possible Applications	Irisin	Human chondrocytes	Cartilage, bone	Improved extracellular matrix synthesis, improved Chondrogenic differentiation	ERK Phosphorylation, Irisin
Carmina, 2023	Semaglutide Treatment of Excessive Body Weight in Obese PCOS Patients Unresponsive to Lifestyle Programs	Semaglutide	Human, obese polycystic ovary Syndrome patients	Systemic	Weight loss, better glucose metabolism	Metabolism, incretin
Sun, 2023	The development of novel multifunctional drug system 7,8-DHF@ZIF-8 and its potential application in bone defect healing	7,8-DHF@ZIF-8, 7,8-Dihydroxyflavone	MC3T3-E1 and HUVEC cells	Bone, vessels	Improved osteogenesis and angiogenesis	BDNF
Lee, 2022	Limonium tetragonum Promotes Running Endurance in Mice through Mitochondrial Biogenesis and Oxidative Fiber Formation	Limonium tetragonum	Mice	Skeletal muscle	Enhanced exercise endurance, increased oxidative fibers, increased mitochondrial content	Mitochondrial metabolism, PKA–CREB–PGC1alpha

Palma-Flores, 2023	(-)-Epicatechin modulates the expression of myomiRs implicated in exercise response in mouse skeletal muscle	(-)-Epicatechin	Mice	Skeletal muscle	Increased fiber size	MyomiRs
Casso, 2022	Initiation of 3,3-dimethyl-1-butanol at midlife prevents endothelial dysfunction and attenuates in vivo aortic stiffening with ageing in mice	3,3-dimethyl-1-butanol	Aging mice	Vessels	Improved vascular endothelial function	NO, ROS
Song, 2022	Sirt6 reprograms myofibers to oxidative type through CREB-dependent Sox6 suppression	MDL-801 (sirt6 deacetylation activator)	Mice	Skeletal muscle	Enhanced endurance performance, increased oxidative fibers and mitochondrial oxidative capacity	Mitochondrial metabolism, Sirt6
Rebalka, 2022	Multi-Ingredient Supplement Supports Mitochondrial Health through Interleukin-15 Signaling in Older Adult Human Dermal Fibroblasts	Multi-ingredient supplement	Human fibroblasts	Skin	Upregulation of proteins involved in mitochondrial function and oxidative phosphorylation, improvement of antioxidant activity	Oxidative stress, PPAR-gamma, Il-15
Liu, 2022	d-Allulose Improves Endurance and Recovery from Exhaustion in Male C57BL/6J Mice	d-Allulose	Mice	Skeletal muscle, systemic	Improved performance, better insulin sensivity	Metabolism, AMPK, PGC-1alpha
Sanni, 2022	The effects of whole-body vibration amplitude on glucose metabolism, inflammation, and skeletal muscle oxygenation	Whole-body vibration	Human trial, female and male adults	Skeletal muscle, systemic	Increased muscle oxygenation, improved glucose metabolism	Myokines, IL-6
Yardimci, 2022	Irisin Improves High-Fat Diet-Induced Sexual Dysfunction in Obese Male Rats	Irisin	Obese rats	Reproductive organs	Better sexual performance, improved sperm morphology and motility, reduced testicular damage	Myokines, Irisin
Pan, 2022	Trehalose ameliorates autophagy dysregulation in aged cortex and acts as an exercise mimetic to delay brain aging in elderly mice	Trehalose	Aged mice	Brain	Improved learning and memory	AMPK, TOR, Autophagy
Hussain, 2022	Infrared sauna as exercise-mimetic? Physiological responses to infrared sauna vs exercise in healthy women: A randomized controlled crossover trial	Infrared sauna	Human trial, women	Cardiorespiratory, cardiovascular system	Increased temperature,	Thermogenesis

Ericsson, 2021	AMPK activator O304 improves metabolic and cardiac function, and exercise capacity in aged mice	O304, pan-AMPK activator	Aging mice	Cardiac system, systemic	Prevention of insulin resistance, improved cardiac function,	Metabolism, AMPK
Momenzadeh, 2021	Muscles proteome analysis; irisin administration mimics some molecular effects of exercise in quadriceps muscle	Irisin	Mice	Skeletal muscle	Differential expression of muscle proteins	Myokines
Lee, 2021	Chronic AdipoRon Treatment Mimics the Effects of Physical Exercise on Restoring Hippocampal Neuroplasticity in Diabetic Mice	AdipoRon	Diabetic rats	Brain	Restoration of Hippocampal Neuroplasticity	Adiponectin, BDNF, AMPK, PGC-1alpha
Martínez-Damas, 2021	Epicatechin treatment generates resilience to chronic mild stress-induced depression in a murine model	Epicatechin	Mice with chronic mild stress	Central Nervous system, skeletal muscle	Resilience to depression	kynurenine aminotransferases, PGC-1alpha-PPAR-delta/alpha
Komiya, 2021	Dietary Olive Oil Intake Improves Running Endurance with Intramuscular Triacylglycerol Accumulation in Mice	Olive Oil	Mice	Skeletal muscle	Improved running endurance, increased muscle triacylglycerol	Metabolism, DGAT1
McDonald, 2021	An Exercise Mimetic Approach to Reduce Poststroke Deconditioning and Enhance Stroke Recovery	Resveratrol	Rats, stroke induced model	Brain, skeletal muscle	Better capillary density in the ipsilesional hemisphere, mitigation of stroke-induced muscle fiber changes	Sirtuins
James, 2021	Timing of acute passive heating on glucose tolerance and blood pressure in people with type 2 diabetes: a randomized, balanced crossover, control trial	Passive heating	Human trial, T2DM patients	Systemic	Increase of eHSP70 concentration and total energy expenditure	eHSP70
Meng, 2020	An extract of Lycium barbarum mimics exercise to improve muscle endurance through increasing type IIa oxidative muscle fibers by activating ERRγ	Lycium barbarum extract	Mice, C2C12 cells	Skeletal muscle	Increase of muscle mass and endurance, switch from glycolytic to oxidative metabolism	Metabolism, ERR-gamma, Sirtuins, PGC-1alpha/beta
Norikura, 2020	cis-Banglene, a bangle (Zingiber purpureum)-derived bioactive compound, promotes mitochondrial biogenesis and glucose uptake by activating the IL-6/AMPK signaling pathway in C2C12 skeletal muscle cells	cis-Banglene	C2C12 cells	Skeletal muscle	Improved glucose uptake, improve mitochondrial biogenesis	Myokines, Metabolisms, IL-6, AMPK

Cao, 2020	Combination of Exercise Training and SOD Mimetic Tempol Enhances Upregulation of Nitric Oxide Synthase in the Kidney of Spontaneously Hypertensive Rats	Tempol (SOD mimetic)	Hypertensive rats	Kidney	Increased NO synthase activity	eNOS
McDonald, 2021	(-)-Epicatechin induces mitochondrial biogenesis and markers of muscle regeneration in adults with Becker muscular dystrophy	Epicatechin	Human trial, Becker dystrophy patients	Skeletal muscle	Modulation of skeletal muscle protein expression, better mitochondrial morphology	Regeneration
Betik, 2021	Whole-Body Vibration Stimulates Microvascular Blood Flow in Skeletal Muscle	Whole-Body Vibration	Human trial, young healthy adults	Skeletal muscle	Improved microvascular blood flow	Thermogenesis
Brunt, 2020	Trimethylamine-N-Oxide Promotes Age-Related Vascular Oxidative Stress and Endothelial Dysfunction in Mice and Healthy Humans	3,3-dimethyl-1-butanol	Aging mice, in vitro carotids	Vessels	Reversed age-related endothelial dysfunction, normalization of oxidative stress and NO bioavailability	NO synthase
Chang, 2020	Irisin prevents dexamethasone-induced atrophy in C2C12 myotubes	Irisin	C2C12 cells	Skeletal muscle	Attenuation of dexamethasone induced atrophy	Myokines, Irisin
Ozemek, 2020	Effects of resveratrol or estradiol on postexercise endothelial function in estrogen-deficient postmenopausal women	Estradiol, resveratrol	Human trial, post menopausal women	Vessels	Enhanced basal endothelial function	Estrogen Receptors
Kim, 2020	Indoprofen prevents muscle wasting in aged mice through activation of PDK1/AKT pathway	Indoprofen	Atrophic mice, C2C12	Skeletal muscle	Activation of oxidative metabolism, increased muscle mass	Metabolism, AMPK
Storlino, 2020	Irisin Prevents Disuse-Induced Osteocyte Apoptosis	Irisin	Hindlimb suspended mice, MLO-Y4 mouse osteocytes	Bone	Inhibition of apoptosis	Myokines, Apoptosis, Erk1/Erk2, caspase 9/3
Kang, 2019	Effects of ursolic acid on muscle mass and bone microstructure in rats with casting-induced muscle atrophy	Ursolic acid	Hindlimb immobilized rats	Skeletal muscle, bone	Improved muscle mass and bone density	No suggestions
Garf, 2019	Complementary Immunometabolic Effects of Exercise and PPARβ/δ Agonist in the Context of Diet-Induced Weight Loss in Obese Female Mice	GW0742 (PPARβ/δ agonist)	Mice	Lymphoid tissue, skeletal muscle, systemic	Weight loss, visceral fat mass reduction, better insulin sensitivity, reduced inflammation	Metabolism, AMPK

Kim, 2019	The mitochondrial-derived peptide MOTS-c is a regulator of plasma metabolites and enhances insulin sensitivity	MOTS-c	Obese mice	Systemic	Variation of metabolic profiles	Metabolism, mitochondria
Yu, 2019	Whole Body Vibration-Induced Omental Macrophage Polarization and Fecal Microbiome Modification in a Murine Model	Whole Body Vibration	Obese mice	Adipose tissue, immune system, gut, systemic	Altered macrophage polarization, gut microbiome	No suggestions
Tran, 2018	A multi-ingredient athletic supplement disproportionately enhances hind leg musculature, jumping performance, and spontaneous locomotion in crickets (Acheta domesticus)	Multi-ingredient supplement	Aging crickets	Locomotor system	Improved mean survivorship, improved morphological properties, improved jumping	No suggestion
Muise, 2019	Pharmacological AMPK activation induces transcriptional responses congruent to exercise in skeletal and cardiac muscle, adipose tissues and liver	Small molecule activators of AMPK	Obese mice and rats	Skeletal muscle, heart, liver, adipose tissue	Better glucose tolerance, improved glucose accumulation and glycogen mobilization, better fatty acid oxidation	Metabolism, AMPK
Linecker, 2020	Exercise Improves Outcomes of Surgery on Fatty Liver in Mice: A Novel Effect Mediated by the AMPK Pathway	AICAR	Mice with steatosis	Liver, systemic	Improved hepatic metabolism	Metabolism, AMPK
Kim, 2018	Concurrent treatment with ursolic acid and low-intensity treadmill exercise improves muscle atrophy and related outcomes in rats	Ursolic acid	Hindlimb immobilized rats	Skeletal muscle	Improvement of atrophied muscle mass, reduction of atrophic genes expression	Atrophy, Murf-1, Atrogin-1
Das, 2018	Impairment of an Endothelial NAD(+)-H(2)S Signaling Network Is a Reversible Cause of Vascular Aging	nicotinamide mononucleotide	Aging mice, C2C12 cells	Skeletal muscle, vessels	Angiogenesis promotion	Metabolism, Sirt-1
Xing, 2018	Local Injections of Superoxide Dismutase Attenuate the Exercise Pressor Reflex in Rats with Femoral Artery Occlusion	Tempol (SOD mimetic)	Rats with femoral artery occlusion	Cardiovascular system, nervous system	Reflex improvement	Oxidative stress
Parrini, 2017	Aerobic exercise and a BDNF-mimetic therapy rescue learning and memory in a mouse model of Down syndrome	7,8-dihydroxyflavone (BDNF-mimetic)	Down syndrome mice model	Brain	Improved brain plasticity, associative learning	BDNF
Molinari, 2017	The mitochondrial metabolic reprogramming agent trimetazidine as an 'exercise mimetic' in cachectic C26-bearing mice	Trimetazidine	Mice with C26 colon carcinoma	Skeletal muscle	Increased grip strength, reduced blood glucose, improved cross-sectional area, activation of trophic	Metabolism, PGC1-alpha

					pathways, induction of mitochondrial biogenesis, oxidative metabolism, angiogenesis	
Paré, 2017	Chronic 5-Aminoimidazole-4-Carboxamide-1-β-d-Ribofuranoside Treatment Induces Phenotypic Changes in Skeletal Muscle, but Does Not Improve Disease Outcomes in the R6/2 Mouse Model of Huntington's Disease	AICAR	Huntington's Disease mouse model	Skeletal muscle, brain	Improved muscle phenotype,	Metabolism, AMPK
Stockinger, 2017	Caloric Restriction Mimetics Slow Aging of Neuromuscular Synapses and Muscle Fibers	Resveratrol, metformin	Old mice, C2C12 cells	Skeletal muscle	Better skeletal musle morphology and neuromuscular junction structure	No suggestions
McGee-Lawrence, 2017	Whole-Body Vibration Mimics the Metabolic Effects of Exercise in Male Leptin Receptor-Deficient Mice	Whole-Body Vibration	Obese mice	Skeletal muscle, liver, bone, systemic	Reduced muscle atrophy, better glycemic control and insulin sensitivity, reduced hepatic steatosis	No suggestions
Bowser, 2017	High-molecular-weight cocoa procyanidins possess enhanced insulin-enhancing and insulin mimetic activities in human primary skeletal muscle cells compared to smaller procyanidins	Cocoa procyanidins	Human primary skeletal muscle cells	Skeletal muscle	Improved glucose uptake and glycogen synthesis	Metabolism, AKT
Ponnusamy, 2017	Pharmacologic activation of estrogen receptor β increases mitochondrial function, energy expenditure, and brown adipose tissue	beta-LGND2 (ER-beta-ligand)	Obese mice	Adipose tissue, systemic	Browning of adipose tissue, increased mitochondrial gene expression, energy expenditure and oxphos	Metabolism, ER
Seferos, 2016	Hypericum perforatum L. treatment restored bone mass changes in swimming stressed rats	Hypericum perforatum L.	Swim stressed rats	Bone, systemic	Better testosterone levels, better bone specific weight and mass density	No suggestions
Neidert, 2016	The serine protease, dipeptidyl peptidase IV as a myokine: dietary protein and exercise mimetics as a stimulus for transcription and release	Whey protein	Human trial, rat, C2C12 cells	Skeletal muscle		
Tomcik, 2017	Fenugreek increases insulin-stimulated creatine content in L6C11 muscle myotubes	Fenugreek	L6C11 cells	Skeletal muscle	Increased total creatine, modulation of protein expression	Metabolism, Insulin

Cerveró, 2016	Chronic Treatment with the AMPK Agonist AICAR Prevents Skeletal Muscle Pathology but Fails to Improve Clinical Outcome in a Mouse Model of Severe Spinal Muscular Atrophy	AICAR	SMA mouse model	Skeletal muscle, nervous tissue	Improved skeletal muscle atrophy and neuromuscular junctions, no effects on motoneuron glutamatergic synapse and on microglial and astroglial reaction.	Metabolism, PGC-1alpha
Marcinko, 2015	The AMPK activator R419 improves exercise capacity and skeletal muscle insulin sensitivity in obese mice	R419 (N-(1-(4-cyanobenzyl)piperidin-4-yl)-6-(4-(4-methoxybenzoyl)piperidine-1-carbonyl)nicotinamide)	AMPK-MKO mice	Skeletal muscle, systemic	Improved insulin sensitivity, improved exercise capacity	Metabolism, AMPK
Brestoff, 2015	Manganese [III] Tetrakis [5,10,15,20]-Benzoic Acid Porphyrin Reduces Adiposity and Improves Insulin Action in Mice with Pre-Existing Obesity	MnTBAP (Manganese [III] Tetrakis [5,10,15,20]-Benzoic Acid Porphyrin)	Obese mice	Skeletal muscle, adipose tissue, systemic	Alteration of energy homeostasis, improvement of insulin action	Metabolism, PKB
Liu, 2015	Neuroprotective effects of an oxyntomodulin analogue in the MPTP mouse model of Parkinson's disease	D-Ser2-oxyntomodulin	mice Parkinson disease model,	Nervous system	Better motor impairment	Glucagone, oxyntomodulin
Kim, 2016	Conjugated linoleic acid (CLA) influences muscle metabolism via stimulating mitochondrial biogenesis signaling in adult-onset inactivity induced obese mice	Linoleic acid	N2KO obese mice	Skeletal muscle	Body weight reduction, better voluntary movement, better mitochondrial biogenesis	Metabolism, AMPK-alpha, PPAR-gamma
Guerrieri, 2015	Exercise-mimetic AICAR transiently benefits brain function	AICAR	Mice	Skeletal muscle, brain	Better synaptic plasticity, cell proliferation, gene expression, oxidative stress	Metabolism, AMPK, Myokines
Zhang, 2015	Neuroprotective effects of (Val8)GLP-1-Glu-PAL in the MPTP Parkinson's disease mouse model	(Val8)GLP-1-Glu-PAL	Parkinson disease mouse model	brain	Improvement of motor impairments, reduction of neuronal loss and apoptosis	Glucagone, apoptosis
Zhou, 2015	Dihydromyricetin stimulates irisin secretion partially via the PGC-1α pathway	Dihydromyricetin	Human trial, rats, L6 myotubes	Skeletal muscle, systemic	Higher irisin levels	Myokines, PGC1-alpha

Dong, 2015	Lung endothelial barrier protection by resveratrol involves inhibition of HMGB1 release and HMGB1-induced mitochondrial oxidative damage via an Nrf2-dependent mechanism	Resveratrol	Mice, mouse MLVEcs	Lung endothelium	Attenuation of oxidative damage, better endothelial permeability and lung histomorphology	Oxidative stress, Nfr-2
Dong, 2015	Pharmacokinetics and pharmacodynamics of PF-05231023, a novel long-acting FGF21 mimetic, in a first-in-human study	PF-05231023 (fibroblast growth factor 21 analogue)	Human trial, T2DM patients	Systemic	triglyceride, total cholesterol and low-density lipoprotein cholesterol	FGF21
Sadasivuni, 2014	CNX-013-B2, a unique pan tissue acting rexinoid, modulates several nuclear receptors and controls multiple risk factors of the metabolic syndrome without risk of hypertriglyceridemia, hepatomegaly and body weight gain in animal models	CNX-013-B2 (Rexinoid)	Obese mice	Skeletal muscle, adipose tissue, liver	Improve insulin sensitivity and glucose tolerance, better body weight, alteration of gene expression	Metabolism, PPAR-alpha, beta, delta
Koba, 2014	Central command dysfunction in rats with heart failure is mediated by brain oxidative stress and normalized by exercise training	Tempol	Rat model of cardiac infarction	Brain	Improvement of central command dysfunction	Oxidative stress
Colaianni, 2014	Irisin enhances osteoblast differentiation in vitro	Irisin	Osteoblasts	Bone	Enhanced differentiation	Myokines
Olesen, 2014	Exercise training, but not resveratrol, improves metabolic and inflammatory status in skeletal muscle of aged men	Resveratrol	Human Trial	Skeletal muscle	No effect	Metabolism
Sun, 2013	Ginsenoside Rg3 improves cardiac mitochondrial population quality: mimetic exercise training	Ginsenoside Rg3	Rats	Cardiac system	improved cardiac adaptations and mitochondrial homeostasis	Metabolism, PGC-1alpha, Nrf-2
E L, 2013	Lactate administration reproduces specific brain and liver exercise-related changes	Lactate	Mice	Brain, liver, systemic	Better weight, glucose and insulin levels, variation of gene expression of mitochondrial genes	Metabolism, PGC-1alpha, VEGF-A
Goh, 2014	Effects of resveratrol in patients with type 2 diabetes mellitus on skeletal muscle SIRT1 expression and energy expenditure	Resveratrol	Human trial, T2DM patients	Skeletal muscle, systemic	Variation of protein expression, better energy expenditure	Metabolism, Sirt-1
Sánchez, 2013	Free fatty acid effects on myokine production in combination with exercise mimetics	Free fatty acids, adrenaline, AICAR	C2C12 cells	Skeletal muscle	Modulation of Il-15 and 6 expression	Metabolism, Myokines

Jahnke, 2012	Metabolic remodeling agents show beneficial effects in the dystrophin-deficient mdx mouse model	AICAR, GW501516	Mdx mice	Skeletal muscle	Influence on body weight and animal activity, increased oxidative capacity, satellite cell activation, better muscle fibrosis	Metabolism, PGC-1alpha
Jeong, 2012	Chitooligosaccharide induces mitochondrial biogenesis and increases exercise endurance through the activation of Sirt1 and AMPK in rats	Chitooligosaccharide	Rats	Skeletal muscle	Increased mitochondrial content, improved exercise endurance	Metabolism, AMPK, PGC-1alpha, Sirt1
Saxena, 2012	Augmentation of aerobic respiration and mitochondrial biogenesis in skeletal muscle by hypoxia preconditioning with cobalt chloride	Cobalt chloride	Rats	Skeletal muscle	Increased mitochondrial biogenesis, glucose uptake and aerobic respiration in skeletal, increased physical performance	Hypoxia, HIF-1-alpha
Nogueira, 2011	(-)-Epicatechin enhances fatigue resistance and oxidative capacity in mouse muscle	(-)-Epicatechin	Aging mice	Skeletal muscle, cardiac tissue	Better physical performance, regulation of oxidative phosphorylation complexes, improved mitochondrial quantity and morphology	Metabolism, Oxidative stress
McCord, 2011	Tempol attenuates the exercise pressor reflex independently of neutralizing reactive oxygen species in femoral artery ligated rats	Tempol, Tiron	Rats with femoral artery occlusion	Cardiovascular system, nervous system	Attenuation of the pressor reflex	No suggestions
Momken, 2011	Resveratrol prevents the wasting disorders of mechanical unloading by acting as a physical exercise mimetic in the rat	Resveratrol	Rats, hind limb unloading	Skeletal muscle, adipose tissue, bone, cardiovascular system, systemic	Prevention of muscle atrophy and loss of function, oxidative capacity maintenance and improved oxidative stress, prevention of bone demineralization	Metabolism, PGC-1alpha, Sirt1
Kumar, 2011	Cordyceps sinensis promotes exercise endurance capacity of rats by activating skeletal muscle metabolic regulators	Cordyceps sinensis	Exercised and non-exercised rats	Skeletal muscle, systemic	Improvement of endurance capacity, better glucose transport, better angiogenic and antioxidant response	Metabolism, AMPK, PGC-1alpha

Bernardo, 2010	Postnatal PPARdelta activation and myostatin inhibition exert distinct yet complimentary effects on the metabolic profile of obese insulin-resistant mice	GW501516, PF-879 (Myostatin antibody)	Obese mice	Skeletal muscle, adipose tissue, liver, systemic	Changes in body weight, fat mass and lean mass, better mitochondrial activity and fiber size, better lipid profiles, improved physical activity	Metabolism, PPAR-gamma; Myokines, myostatin
Saxena, 2010	Hypoxia preconditioning by cobalt chloride enhances endurance performance and protects skeletal muscles from exercise-induced oxidative damage in rats	cobalt chloride	Rats	Skeletal muscle, systemic	Increased physical performance, improved cellular oxygen sensing, improvement of GSH/GSSG ratio and lipid per oxidation	Oxidative stress
Narkar, 2008	AMPK and PPARdelta agonists are exercise mimetics	GW501516, AICAR	Mice	Skeletal muscle, systemic	Better muscle gene expression, muscle remodeling, increased running endurance	Metabolism, AMPK-alpha, PPAR-delta
Dambrova, 2008	Comparative pharmacological activity of optical isomers of phenibut	Phenibut	Stressed mice and rats	Nervous system	Decreased immobility time, increased analgesia	gamma-aminobutyric acid
Nelson, 2007	The incretin mimetic exenatide as a monotherapy in patients with type 2 diabetes	Exenatide	Human trial, T2DM patients	Systemic	Reduction of glycosylated hemoglobin, better fasting plasma glucose, better body weight	No suggestions
Tharakan, 2006	Trichopus zeylanicus combats fatigue without amphetamine-mimetic activity	Trichopus zeylanicus	Rats, Dwarf Ames mice	Skeletal muscle, systemic	Anti-fatigue effect	No suggestions
Fehse, 2005	Exenatide augments first- and second-phase insulin secretion in response to intravenous glucose in subjects with type 2 diabetes	Exenatide	Human trial, T2DM patients	Systemic	Restoration of insulin secretory pattern	Incretins
Poon, 2005	Exenatide improves glycemic control and reduces body weight in subjects with type 2 diabetes: a dose-ranging study	Exenatide	Human trial, T2DM patients	Systemic	Reduction of glycosylated hemoglobin, better fasting plasma glucose, better body weight	Incretins
Zhang, 2004	Redox modulation of the liver with chronic antioxidant enzyme mimetic treatment prevents age-related oxidative damage associated with environmental stress	EUK-189 (SOD/catalase mimetic)	Aged rats	Liver	Improved GS/GSSG ratios, protection from liver injury	Oxidative stress

Truel, 1998	Sodium valproate reduces immobility in the behavioral 'despair' test in rats	Sodium valproate	Rats	Nervous system
